# DARPins: Promising Scaffolds for Theranostics

**DOI:** 10.32607/20758251-2019-11-4-42-53

**Published:** 2019

**Authors:** O. N. Shilova, S. M. Deyev

**Affiliations:** Shemyakin–Ovchinnikov Institute of Bioorganic Chemistry, Russian Academy of Sciences, Moscow,

**Keywords:** DARPin, targeted therapy, barnase

## Abstract

Monoclonal antibodies are the classical basis for targeted therapy, but the development of alternative binding proteins has made it possible to use non-immunoglobulin proteins as targeting modules. The advantages of DARPins, scaffold proteins based on ankyrin repeats, over antibodies are as follows: small size, stability over a wide range of temperatures and pH values, low aggregation tendency, and ease of production in heterologous expression systems. The differences in the structure of the paratope of DARPin and antibodies broaden the spectrum of target molecules, while the ease of creating hybrid fusion proteins allows one to obtain bispecific and multivalent constructs. In this article, we summarize recent data on the development of therapeutic and imaging compounds based on DARPins.

## INTRODUCTION

The hybridoma technology described by Kohler and Milstein in 1975 [1] has enabled the production of monoclonal antibodies, which are used in research and diagnostics, as well as in therapy. Due to their high affinity and specificity, monoclonal antibodies have become the "magic bullet" underlying targeted therapy. The first therapeutic monoclonal antibodies were acquired in 1986. To date, 82 monoclonal antibodies have been approved for clinical use by the Food and Drug Administration (FDA), and the number of approved monoclonal antibodies continues to grow. However, antibodies have some disadvantages: their relatively large size (150 kDa) can limit diffusion in both normal tissues [2] and solid tumors [3]; the Fc region prolongs the time of blood circulation, but it can also cause unwanted effects [4]. In addition, full-length antibodies require complex folding and specific glycosylation and, therefore, have to be produced in mammalian cells, which makes them expensive. Another problem arises from the homology between murine and human proteins, which complicates obtaining antibodies specific to conserved proteins.

Many of the aforementioned problems have been solved by obtaining shortened and single-chain antibodies. The development of recombinant antibody technology has led to the replacement of conventional immunization with fully synthetic libraries free of the restrictions on the autospecificity that is typical of lymphocytes. Later, methods for molecule selection based on its affinity to a ligand were used for other proteins, making antibodies dispensable [5]. In 2018, the importance of these findings was recognized with the Nobel Prize in Chemistry "for the directed evolution of enzymes and binding proteins." Half of the prize was awarded to American bioengineer Frances H. Arnold "for the directed evolution of enzymes," and the other half was awarded to George P. Smith and Sir Gregory P. Winter "for the phage display of peptides and antibodies." With the help of these technologies in the past 20 years, a variety of alternative scaffolds have been developed, including monobodies (derived from fibronectin type III), anticalins (derived from lipocalins), affibodies (derived from immunoglobulin-binding protein A), and DARPins (derived from ankyrin repeats). Like antibodies, these proteins usually have a "constant" scaffold and "variable" sites in which amino acid substitutions do not alter the protein conformation [6]. Designing alternative scaffolds involves two stages: (1) the design of a library of protein variants by random site-specific mutagenesis and (2) selection of molecules using phage, ribosome, or yeast display, linking genotype (a protein gene sequence) and phenotype (its ability to bind to the target).

The advantages of these alternative binding proteins include their small size, which facilitates tumor penetration; the absence of Fc-avoiding antibody-mediated cytotoxicity and complement-mediated cytotoxicity; in many cases, high thermostability that enables long-term storage of a preparation at room temperature without loss of activity; ease of production in bacteria, and even the possibility of performing direct chemical synthesis.

**Fig. 1:**
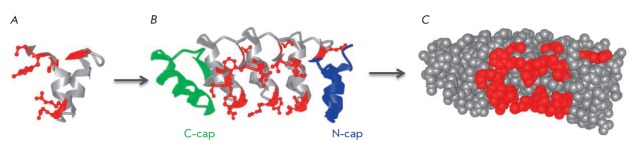
The structure of DARPins. *A *– the structure of the consensus ankyrin repeat; the constant part is shown in gray; the variable regions are shown in red. *B *– the structure of a DARPin molecule. Two or three binding motifs form the binding surface through variable amino acids (shown in red); the hydrophobic regions are shielded by the C-cap and N-cap. *C *– 3D structure of a DARPin molecule, the variable amino acids are shown in red

Many types of alternative scaffolds are based on proteins with repeating motifs: leucine-rich repeats (LRRs), ankyrin repeats (ARs), Armadillo repeats (Arms), and tetratricopeptide repeats (TPRs). Repeat-based proteins are actively used, because they have a long binding surface whose size can be varied and a rigid scaffold formed by the "constant" regions [7]. This paper focuses on DARPins, which are artificial proteins that are based on ankyrin repeats. In eukaryotic cell proteins built from ankyrin, repeats bind to a variety of targets, providing cytoskeletal organization and regulation of enzyme activity [8]. The natural variety of these proteins was used to create a consensus motif containing variable regions and able to combine with neighboring motifs to form a single rigid structure (*Fig. 1 A, B*).

## USING THE STRUCTURAL FEATURES OF DARPins IN BASIC RESEARCH AND BIOTECHNOLOGY

DARPins can be utilized as binding molecules in most technologies using shortened variants of monoclonal antibodies. However, DARPins have other beneficial properties in addition to their small size. The ease of production in bacteria allows one to create fusion proteins and add sequences for purification and labeling, while the absence of cysteine residues in the DARPin molecule allows one to introduce a unique additional cysteine for precise conjugation.

DARPins consist of tightly packed ankyrin repeats, each forming a β-turn and two antiparallel α-helices. A single repeat typically consists of 33 amino acids, six of which form the binding surface. During recombinant library design, these sites are used to introduce the codons of random amino acids, except for cysteine (to avoid the formation of disulfide bonds), as well as glycine and proline (since some amino acids are part of the α-helix) [6]. DARPins are typically formed by two or three of the binding motifs contained between the N- and C-terminal motifs shielding the hydrophobic regions *(Fig. 1)*. DARPins are small proteins (14–18 kDa) that are extremely thermostable (their melting point (Tm) can reach 90°C) and resistant to proteases and denaturing agents. They can be produced in bacteria with a high yield of up to 200 mg of protein from 1 liter of liquid culture [6].

Both ends of the DARPin polypeptide chain form α-helices, facilitating the design of geometrically precise multimers. Thus, the molecular "clamp" wrapping the GFP molecule forming a stable but reversible complex has been created based on two DARPins that recognize different but overlapping epitopes of the green fluorescent protein (GFP) by computer simulations. Such clamps were used for the oriented covering of a sensor chip for surface plasmon resonance with proteins fused to GFP and for chromatographic purification of such proteins on sepharose conjugated to this diDARPin. DiDARPins conjugated to a fluorescent dye amplified the signal from rare GFP-labeled proteins on the cell surface and allowed a more accurate detection of these cells by flow cytometry [9].

A DARPin forming a trimer through the trimerizing motif added to it was created using computer modeling. The obtained DARPin binds to the trimeric protein of adenovirus serotype 5 (Ad5). This protein was shown to be able to almost irreversibly bind to the adenovirus capsid. Adding one more DARPin specific to the target cell receptor enabled efficient infection of cells expressing the corresponding tumor marker (HER2, EGFR or EpCAM) [10].

The rigidity and small size of DARPins made it possible to create dimers that affect signaling of extracellular receptors through fixation of receptors in certain conformations or bringing close molecules that generate competing signals. Utilization of a bivalent DARPin in this way enabled selective suppression of the activity of the mast cells that had bound IgE immune complexes. One of the modules of this dimer recognizes the constant part of IgE in a complex with FcεRI with high affinity; the other module binds to low-affinity FcγRIIB, which exhibits an inhibitory effect on mast cells. This recombinant protein specifically inhibits mast cell degranulation in vivo [11]. A similar approach was applied to create the bispecific diDARPin, which inhibited the proliferation signal from the HER2 receptor and had a cytotoxic effect on HER2-positive cancer cells [12].

Along with multimerization, DARPins can form rigid constructions connected by flexible linkers through introduction of alternative C- and N-terminal motifs sharing a common α-helix. In these di- and trimers, DARPins still retain their ability to simultaneously bind their targets and stabilize them for crystallization [13]. One of such DARPins, which was found to improve the crystallization of its partners, was used to create rigid dimers with DARPin specific to JNK1, which allowed the researchers to obtain crystals of these complexes and reveal the structural features explaining the specificity of DARPins to the kinase isoform and their ability to inhibit its activity [14].

The disadvantages of DARPins as binding modules include their concave binding surface, rigidity, and incomplete randomization of amino acid residues in variable sites, which could potentially limit the range of possible targets. However, these limitations can be overcome: LoopDARPins, a new generation of DARPins, has been created for this purpose. In LoopDARPins, the central β-turn is replaced with a larger convex H3 loop from the immunoglobulin molecule. This insert made it possible to change the geometry of the antigen-binding surface and introduce a flexible motif with a higher amount of variable amino acid residues, as well as improve binding selectivity [15].

However, the concave binding surface of DARPins can also become an advantage. Another DARPin feature (namely, the absence of cysteine residues in the protein that allows introduction of a single cysteine near the surface of interaction with the target and using it for conjugation) makes it possible to take advantage of this drawback. In a study by Kummer et al. [16], DARPin specific to the phosphorylated form of ERK (pERK) was conjugated to an environment-sensitive merocyanine dye: the intensity of its fluorescence increases in a hydrophobic environment; i.e., when DARPin binds to pERK. Hence, a biosensor for detecting ERK phosphorylation was obtained. Since it was shown that DARPin does not itself recognize phosphate but detects changes in the conformation of the activation loop [17], this approach can be used for other proteins that change their conformation during functioning.

Therefore, even the relative disadvantages of DARPins can be used to create unique constructs. However, the advantages of DARPins have made it possible to find many uses for these proteins, primarily in therapy and the diagnosis of cancer.

## APPLICATIONS OF DARPins IN CANCER DIAGNOSIS AND THERAPY

The principles for DARPin design were described in 2003 [18]. In 2007, this technology was applied to obtain high-affinity proteins that bind to the HER2 tumor marker [19]. Later, DARPins binding to other molecules involved in carcinogenesis were obtained. The targets included EpCAM [20], EGFR [21], VEGF [22], HGF [22], cathepsin B [23], KRAS [24], etc. However, to date, the majority of targeted agents are based on HER2-binding DARPins. This can be explained by the therapeutic significance of the target. The HER2 (ErbB2) protein is a tyrosine kinase receptor with a low level of expression on the surface of human epithelial cells. HER2 is normally involved in various intracellular signaling pathways but mainly stimulates the HER3/PI3K/Akt pathway and mitogen-activated protein kinase (MAPK) cascade [25], leading to cell proliferation. The HER2 antigen is overexpressed in 20–30% of mammary gland and ovary tumors and bolsters the aggressive properties of the tumor. That is why the standard diagnostic protocols for breast cancer involve determining the HER2 expression level [26]. *ERBB2 *gene amplification can also be observed in gastric and intestinal adenocarcinomas [27], carcinomas of the ovary [28], endometrium [29], prostate gland [30], as well as the salivary glands, vagina, cervix and the bladder [31]. Two murine humanized antibodies are currently used in HER2-positive cancer therapy: trastuzumab (Herceptin, Roche-Genentech) binding to subdomain IV of HER2 and pertuzumab (Perjeta, Roche-Genentech), which binds to subdomain II of the receptor [32]. In addition, trastuzumab conjugated with the microtubule assembly inhibitor (trastuzumab-emtazine, Kadcyla, Roche) [33] and two chemical tyrosine kinase domain inhibitors are used: lapatinib (Tykerb or Tyverb, GlaxoSmithKlein) [34] and neratinib (Nerlynx, Pfizer) [35]. These drugs have been approved for HER2-positive breast cancer, gastric cancer, and gastroesophageal cancer [36]. However, the indications for their use can be expanded in the near future. According to the results of the MY PATHWAY study, a statistically significant response to the trastuzumab and pertuzumab therapy was shown for patients with 9 types of HER2-positive tumors: colorectal cancer (38% of patients), bladder cancer (33%), gallbladder cancer (29%), salivary gland cancer (80%), non-small cell lung cancer (13%), pancreatic cancer (22%), ovarian cancer (13%), prostate cancer, and skin cancer (a single patient in each case) [37]. Therefore, we can conclude that the potential of HER2-specific targeted therapy is not limited to breast cancer and gastric cancer. At the same time, the existing targeted HER2-directed therapy significantly enhances the effectiveness of combination therapy but a complete response or prolongation of patients’ survival to more than 5 years are still rare events, which continues to stimulate the search for novel drugs.

**Fig. 2:**
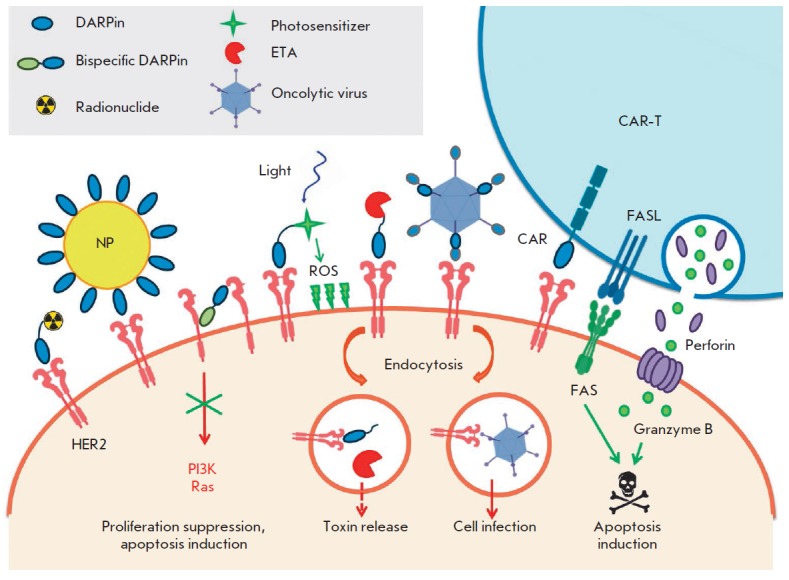
Application of DARPins in cancer cell visualization and elimination. DARPins can inhibit cell signaling molecules, thus suppressing cell proliferation, or serve as targeting modules for the delivery of various agents: radionuclides, nanoparticles or liposomes, photosensitizers, protein toxins, oncolytic viruses, and lymphocytes with chimeric antigenic receptors. HER2 – human epidermal growth factor receptor 2; NP – nanoparticle; ROS – reactive oxygen species; PI3K – phosphoinositide-3-kinase; Ras – small GTPase Ras; CAR – chimeric antigen receptor; CAR-T – T-lymphocyte expressing the chimeric antigen receptor; FAS – death receptor (CD95, APO-1), an inducer of extrinsic apoptosis pathway; FASL – ligand of the FAS receptor (CD95L, CD178); ETA – truncated *Pseudomonas aeruginosa *exotoxin A

*Figure 2* summarizes the main ways of using DARPins for developing agents for cancer diagnosis and treatment.

Tumor imaging is important for conducting preclinical trials of new drugs in animals, for validating patient’s diagnosis, and evaluating therapy efficacy. In animal models, far-red fluorescent proteins, such as mCherry, can be applied to allow intravital visualization of a tumor [38]. Cherry and HER2-specific DARPin 9_29 were fused to obtain the recombinant protein DARPin-mCherry, which specifically stains HER2- positive cancer cells [39] and is used for the functionalization of nanoparticles [40, 41, 42, 43] as described below.

Radionuclides selectively accumulating in the tumor are used for tumor imaging in the human body. Monomeric DARPins can act as binding modules for high-affinity radio immunodiagnostics, in which proteins conjugated to a radionuclide carrier (typically a chelator or quasicovalent technetium complexes) are used [44]. This technology was originally developed for single-chain antibodies, but soon it was applied to other scaffold proteins, since the basic requirements for binding modules for radioimmune diagnostics include high affinity and small size [45, 46]. DARPins have both of these properties and can be successfully utilized for the radioactive imaging of tumors. For example, HER2-specific DARPins G3 and 9_29 were used for obtaining conjugates with the desired pharmacokinetics and reduced accumulation in the liver [47, 48, 49]. As for cancer therapy, DARPins can be used both for the delivery of toxic modules and for the inhibition of cell signaling pathways thanks to the specific binding of membrane receptors. A bispecific DARPin dimer with a linker of a certain length was shown to fix the extracellular parts of neighboring HER2 receptors in a nonfunctional conformation that does not allow them to form dimers and transduce mitogenic signals, which had cytostatic and cytotoxic effects on HER2-dependent cancer cells [12]. The dimer was used to design the tetrameric MP0274 drug: it consists of modules recognizing the domains I and IV of the HER2 receptor and two modules that bind to human serum albumin, which increase the circulation time of the protein in the blood. The first phase of clinical trials of this drug was started in 2017 [50].

Clinical trials are underway for MP0250, another multivalent DARPin. One polypeptide chain of this protein contains a module that binds to the vascular endothelial growth factor VEGF-A, a module binding to the hepatocyte growth factor HGF, and two modules binding to human serum albumin [22]. Therefore, the drug inhibits two important cancer cell signaling pathways: VEGF/VEGFR and HFG/cMet; its binding to albumin ensures long-term circulation. MP0250 is the first multimeric DARPin tested in patients [51]. In a phase I clinical trial, this drug was well-tolerated at doses sufficient to suppress VEGF activity. In 2018, phase Ib/II clinical trials to evaluate MP0250 in combination with osimertinib for the treatment of patients with nonsquamous non-small cell lung cancer (NSCLC) with EGFR mutations were started [52]. In 2017, phase II clinical trials of MP0250 in combination with bortezomib and dexamethasone for treating patients with refractory and relapsed multiple myeloma (RRMM) were initiated [53].

Another way to create DARPins with tailored pharmacokinetics is conjugation with polyethylene glycol and topical application of the conjugates. One such conjugate, abicipar specific for VEGF, is used for neovascular age-related macular degeneration (ADE) and diabetic macular edema (DME) [54]. This drug is currently undergoing phase III clinical trials.

## DARPin-BASED TUMOR TARGETING TOXINS

The simplicity of DARPin production in the bacterial expression system has stimulated the development of antitumor agents based on protein toxins. *Pseudomonas aeruginosa *exotoxin A (PE, ETA) is one of the most efficient apoptosis inducers thanks to its own enzymatic activity that inhibits translation. PE consists of three domains. Domain I is specific to the α-2-macroglobulin receptor of animal cells (LRP1, CD91) and provides internalization of the toxin molecule into the cell. Domain II contains furin proteolysis sites and disulfide bonds reduced by protein disulfide isomerases, which are thus involved in the intracellular processing of the toxin molecule. Domain III exhibits intrinsic catalytic activity: it ADP-ribosylates eukaryotic eEF2, thereby blocking protein biosynthesis in the cell, ultimately leading to cell death [55]. The domain structure of the exotoxin allows one to use its truncated variants preserving catalytic activity, while the natural binding domain can be replaced with targeting molecules of desired specificity. In this case, it is sufficient that the agent enters the endosome where the effector module is cut off by furin protease and the toxin is transported to the endoplasmic reticulum due to the KDEL retrograde transport signal, and subsequently released into the cytosol from it [56].

EpCAM-specific DARPin Ec4 was used to deliver a truncated *P. aeruginosa *exotoxin to colon cancer cells HT29. The resulting DARPin-ETA protein exhibited an antitumor activity both in vitro and in vivo [57]. ETA was also used to suppress the growth of HER2-positive tumors. Since DARPin 9_29 effectively stimulates the internalization of HER2 into a complex with the protein partner [58], this targeting module is well-suited for delivering exotoxin fragments to cancer cells. The DARPin-PE40 targeted toxin was created using the DARPin 9_29 module and a *P. aeruginosa *exotoxin A fragment with a molecular weight of 40 kDa. It successfully induced apoptosis in HER2-overexpressing cells, exhibited selective in vitro toxicity, and effectively suppressed breast cancer cell growth in a xenograft model [59].

One of the problems related to antitumor agents based on the Pseudomonas exotoxin is high immunogenicity. Being a protein of bacterial origin, ETA causes the formation of neutralizing antibodies, which reduce therapy effectiveness and increase the risk of anaphylactic reactions. Various approaches have been developed to solve this problem: mutagenesis of PE, followed by chemical modification (PEGylation); suppression of the patient’s immune system; as well as detection and elimination of immunodominant epitopes of B and T cells by mutagenesis. The latter of these approaches is the most universal and compatible with different regimens of tumor therapy [60]. DARPin- LoPE containing an exotoxin fragment with deleted or mutant immunodominant epitopes exhibited selective toxicity with respect to HER2-overexpressing cells in vitro at picomolar concentrations [61] and effectively suppressed the growth of ovarian cancer cells in the xenograft model [62]. Moreover, the nonspecific toxicity and immunogenicity of DARPin-LoPE were lower than those of DARPin-PE40: so, the contribution of DARPin to these side effects was negligible.

## APPLICATIONS OF DARPins IN TARGETED PHOTODYNAMIC THERAPY

Photodynamic cancer therapy relies on the use of photosensitizers that convert oxygen into reactive oxygen species (mainly singlet oxygen (1O_2_)) at certain wavelengths [63]. The advantage of photodynamic therapy over chemotherapy consists in smaller exposure of healthy tissues, since only part of the body is irradiated. However, such a localized exposure does not completely prevent side effects, such as sensitization of the skin and the retina.

Two approaches are used to solve this problem: increasing selective accumulation of the photosensitizer in the tumor thanks to the physicochemical properties of the molecule per se and covalent binding of the targeting modules to a photosensitizer (targeted photodynamic therapy) [64]. Monoclonal antibodies were the first targeting molecules used to specifically deliver a photosensitizer to a tumor. This approach was developed after the study by Mew D. et al., who showed that hematoporphyrin can be directly conjugated to a monoclonal antibody specific to the myosarcoma antigen and demonstrated the advantages of the resulting immunoconjugate over hematoporphyrin in vivo [65]. Further development of targeted photodynamic therapy has led to the design of conjugates that include other targeting modules selectively accumulating in the tumor due to the biochemistry of malignant cells and their signaling pathways. For example, the application of the conjugates of photosensitizers with folic acid was proposed for tumors dependent on folic acid. Peptide ligands are also currently being developed; these ligands are a tool for delivering chemical photosensitizers to tumor cells carrying specific integrins and hormone receptors on their surface [66].

The conjugates of antibodies and photosensitizers effectively eliminate cancer cells that carry known surface markers in both in vitro studies and in vivo [67]. However, chemical conjugation of photosensitizers and antibodies has a number of drawbacks, such as low reproducibility of conjugate synthesis, aggregation, the presence of an unconjugated photosensitizer in the preparation, loss of antibody affinity to the receptor, and changes in the physical properties of the photosensitizer [68].

A fundamental solution to these problems is to design genetically encoded hybrid molecules containing both phototoxic and targeting components. This eliminates the need for chemical conjugation of components and enables the production of fused recombinant molecules of constant composition, thus ensuring steadily reproducible functionality. It became possible to produce these photosensitizers after phototoxic proteins capable of producing reactive oxygen species when exposed to light at a specific wavelength were discovered. To date, two types of phototoxic proteins are known. These are the KillerRed [69] and KillerOrange proteins [70], the *Aequorea victoria *GFP derivatives, as well as miniSOG [71] and the miniSOG2 [72] protein, the derivatives of the *Arabidopsis thaliana *phototropin.

DARPin 9_29 was used to deliver the phototoxic miniSOG protein (miniSinglet Oxygen Generator) to cancer cells. This protein is obtained from the LOV2 (Light Oxygen Voltage) domain of phototropin 2 (AtPhot2) by site-specific mutagenesis. The LOV domain contains the flavin mononucleotide (FMN) cofactor, which is excited by blue light, after which the energy of the excited state is consumed for the formation of covalent bonds with conserved cysteine 426. Replacing the cysteine 426 participating in this reaction with glycine has altered protein activity. In response to blue light irradiation, all the energy of the excited state of FMN was spent on singlet oxygen formation. After additional mutagenesis, the variant with a quantum yield of singlet oxygen of 0.47 ± 0.05 was selected. The absorption spectrum of miniSOG contains two peaks at 448 and 473 nm; the fluorescence spectrum peaks correspond to 500 and 528 nm [71].

The miniSOG protein was originally designed as a genetically encoded marker for electron microscopy: miniSOG generates singlet oxygen in quantities sufficient for initiating oxidative polymerization of diaminobenzidine (DAB). The polymer obtained by oxidation of DAB interacts with osmium tetroxide; the product of this reaction is used as a label for electron microscopy. In addition, miniSOG can be used as a toxic module for ontogenesis studies, selective inactivation of proteins, and photodynamic therapy [73, 74, 75].

A genetically encoded 4D5scFv-miniSOG immunophotosensitizer was based on the anti-HER2 mini antibody and the phototoxic protein miniSOG. 4D5scFv-miniSOG selectively destroys HER2-positive SK-BR-3 breast adenocarcinoma cells under irradiation. The cytotoxic effect of 4D5scF-vminiSOG against this cancer cell line is eightfold stronger than the effect of the chemical conjugate of porphyrin with the same targeting module [76]. However, the overproduction of 4D5scFv-miniSOG in bacteria leads to the aggregation of most of the target protein in inclusion bodies, and its renaturation is ineffective. The replacement of the targeting module with HER2-specific DARPin 9_29 helped to solve the problem related to the production of the target protein in bacteria in soluble form; the yield of the protein was 15 mg from 1 liter of liquid culture. DARPin-miniSOG exhibited selective in vitro toxicity against HER2- overexpressing SK-BR-3 breast adenocarcinoma cells [77]. Notably, the fluorescent properties of DARPin-miniSOG allowed one to estimate the rate of internalization and the recycling of the HER2 molecule [58], as well as compare the internalization rates of 4D4scFv and DARPin 9_29 in a complex with this receptor [78]. Nevertheless, other fluorescent modules or dyes are preferred for the visualization of HER2-positive cancer cells, since miniSOG has a relatively low fluorescence quantum yield and the emission spectrum overlaps with cell autofluorescence [79].

DARPins can also be used to deliver phototoxic nanoparticles, enabling the creation of multifunctional antitumor agents, which will be discussed further.

## APPLICATION OF DARPins IN NANOPARTICLE DELIVERY

Nanostructures are increasingly used in basic research, as well as in the diagnosis and therapy of various diseases. Some types of nanoparticles have unique characteristics that make it possible to use them for efficient contrasting of pathogenic foci using X-ray, infrared, and other types of electromagnetic radiation or acoustic waves. Most of the developments have been made in the field of antitumor nanoparticles, primarily due to the fact that imperfect vascularization and disorganization of cell–cell contacts of the tumor make it possible for many types of nanoparticles to penetrate the tumor more efficiently than normal tissue [80, 81]. The advantage of nanoparticles over low-molecular-weight drugs and proteins is that a single agent can have several functions, including particle-targeting to cancer cells using surface modification. Monoclonal antibodies are often used for this purpose. However, the problems related to proper orientation and standardization of the number of antibodies per particle still remain relevant for full-length antibodies [82]. In addition to antibodies and their fragments, other molecules can be used: alternative scaffolds; proteins that are specifically captured by a tumor, such as growth factors and transferrin; aptamers; and low-molecular-weight substances (e.g., folic acid) [56, 57].

Similar to monoclonal antibodies, DARPins can be used to functionalize nanoparticles [83]. DARPin 9_29 was used to deliver upconverting nanoparticles into a tumor during photodynamic therapy. NaYF_4_ : Yb_3_^+^ Tm_3_^+^ / NaYF_4_ particles emitting ultraviolet radiation when exposed to infrared light were coated with the DARPin-mCherry protein [39], which allows visualization of cancer cells thanks to the far-red fluorescent mCherry module [40]. DARPin 9_29 and the DARPin-mCherry protein containing it were also used to coat 5-nm gold nanoparticles [41] and gold nanorods [42]. DARPin was efficiently coupled with the particle surface to form a crown consisting of approximately 35 protein molecules, thus reducing particle aggregation. Notably, DARPin interacted with nanoparticles in a way, leaving its HER2-binding surface free, which ensured selective binding of the resulting nanoparticles to HER2-overexpressing cells [41].

DARPins and DARPin-containing proteins can be successfully coupled with nanoparticles using carbodiimide conjugation. DARPin 9_29 was covalently bound to upconverting radioactive nanoparticles coated with a maleic anhydride and 1-octadecene (PMAO) copolymer. The resulting nanoparticles were used to visualize breast tumors in a xenograft mouse model and exhibited low side toxicity in vivo [84]. The same conjugation method was applied to functionalize upconverting nanoparticles with the DARPin-mCherry protein [43]. DARPin-PE40 was coupled with upconverting radioactive nanoparticles in the same way, making it possible to visualize tumors in vivo and efficiently eliminate HER2-overexpressing cells both in vitro and in vivo [85]. Insertion of unique cysteine residue allowed one to conjugate HER2-specific DARPin G3 with fluorescein maleimide and then to bind the labeled DARPin to superparamagnetic nanoparticles coated with polylactic acid by activating its C-terminal carboxyl groups with carbodiimide [86]. DARPin was also attached to nanostructures via maleimide conjugation. This method was utilized for DARPin 9_29 conjugation with the surface of ETA-containing liposomes functionalized using Trout’s reagent [87].

Hence, the standard methods for immunoglobulin coupling to nanoparticles can be applied to DARPins. However, DARPins can also be embedded into nanostructures in the form of fused proteins that interact with the particle surface. This approach allows one both to achieve the desired orientation of the binding module and to assemble the targeting modules according to the principle of a construction kit. For example, a DARPin-Bn protein consisting of DARPin 9_29, a flexible linker and barnase ribonuclease, was used to create targeted silicon nanoparticles. These nanoparticles are coated with a barstar protein fused to a SiO_2_-binding peptide (SBP-Bs), which attaches SBP-Bs to the particle. As barnase and barstar bind to each other with a very high affinity (*K*_a_ = 10^14^ M^-1^), these proteins allowed one to assembly the outer layer of nanoparticles in a solution without using conjugation or to implement the pre-targeting strategy when the targeted protein was delivered to the cells to which the nanoparticles were subsequently added [88]. Fusion of barnase and the peptide binding to the magnetite surface made it possible to utilize the same DARPin-Bn protein to functionalize magnetite nanoparticles and deliver them to cancer cells [89].

To sum up, DARPins can be used, along with antibodies and their fragments, to create targeted nanoparticles. Moreover, their small size and simplicity of production in bacteria (including fusion proteins) provide unique opportunities for maintaining the affinity and specificity of the binding module thanks to the favorable orientation of the molecule.

## APPLICATION OF DARPins IN DESIGNING ONCOLYTIC VIRUSES

Molecules derived from viruses and bacteria are widely used to obtain antitumor agents [81], but replicative active viral particles can be utilized for tumor cell destruction [90]. Oncolytic viruses form a new, very peculiar class of therapeutic drugs that largely act in the patient’s body on their own. Some viruses have natural tropism to tumor cells, but oncolytic agents are more likely to be based on viruses that can be retargeted by modification of surface proteins (e.g., measles virus, adenovirus, vesicular stomatitis virus, vaccinia virus, and herpes simplex virus) [90]. The natural specificity of the virus can be changed using bispecific adapter proteins, as has been successfully done for adenoviruses using trimerizing DARPins [10]. However, the fusion of targeting modules with envelope proteins is used more often, since in this case all the properties of the virus are encoded by its genome. Like single-chain antibodies, DARPins can be used for such retargeting, and their small size facilitates successful encoding of DARPins sequences in viral vectors.

The measles virus envelope protein was modified by DARPins specific to HER2, EGFR, or EpCAM. The resulting viral particles lost their natural receptors tropism and selectively infected cells, overexpressing the corresponding tumor marker. Viral particles bearing HER2-specific DARPin on the surface caused cell lysis more efficiently than virus functionalized with a HER2-specific single-chain antibody. The use of two DARPin-linked DARPins recognizing HER2 and EpCAM allowed one to create bispecific viral particles that retain the high cytolytic activity of monospecific virions [91, 92].

An adeno-associated virus coated with the DARPin-fused modified envelope protein VP2 was also used to infect HER2-positive cancer cells. The resulting virions specifically infected HER2-positive cells and delivered vectors encoding either the luciferase gene or the herpes simplex virus thymidine kinase gene (HSV-TK) to SK-OV-3 cells *in vivo*. Viral particles containing a gene therapy vector encoding HSV-TK, in combination with ganciclovir, effectively suppressed xenograft tumor growth, without causing hepatotoxicity [93]. Similar viral particles were obtained using EGFR-specific DARPin and affibody, and both agents showed selective toxicity towards EGFR-positive cells *in vitro *[94].

## APPLICATION OF DARPins IN THE DESIGN OF CHIMERIC ANTIGEN RECEPTORS

The accumulated knowledge on the functioning of the immune system allowed us to elaborate the technology of targeted cancer therapy based on cytotoxic lymphocytes: T lymphocytes and NK cells. In this case, the lymphocytes are transduced with constructs that encode the chimeric antigen receptor (CAR), which is specific to the tumor antigen and has all the domains necessary for cell activation, including the signal se quences of the co-stimulating molecules of the natural receptor [95]. When activated through chimeric receptors, lymphocytes secrete proinflammatory cytokines and induce apoptosis in target cells through the extrinsic FAS receptor pathway and with the help of the granzymes that directly activate effector caspases and the caspase-independent pathways of cell death [96]. T cells with a chimeric antigenic receptor (CAR-T) successfully fought chemotherapy-resistant hematologic tumors to ensure complete cure in a large number of patients [97, 98]. Most of the chimeric receptors developed to date contain single-chain antibodies as an antigen-recognizing domain; however, DARPins can also be used as targeting modules for CAR. Moreover, DARPins have some advantages over single-chain antibodies. Thus, they are more compact, meaning that their coding sequences occupy less space in a lymphocyte transducing virus vector. Furthermore, DARPins are more thermodynamically stable. Finally, their binding surface is formed by a single polypeptide, unlike that of the antibodies whose paratope is formed by two immunoglobulin domains originating from different polypeptides. This means that DARPins can be used to obtain multispecific CARs [99].

CAR-T carrying a receptor based on HER2-specific DARPin G3 had the same level of activation as cells with a chimeric receptor containing single-chain antibody FRP5. The DARPin-containing CAR-T exhibited high toxicity against HER2-positive cancer cells and low toxicity against control cells not expressing HER2 [99]. Similar results were obtained when comparing CAR-T therapy based on 4D5 antibody and CAR-T based on DARPins G3 and 9_29. All the studied cell types specifically recognized HER2 and exhibited high cytotoxicity against HER2-positive cells in vitro. Cells with receptors based on DARPin G3 showed the highest efficacy. In the ovarian cancer xenograft model, the differences between CAR-T based on different DARPins were more pronounced: cells with a receptor based on 4D5scFv and DARPin G3 better infiltrated the tumor and more effectively suppressed its growth [100]. Generally, a conclusion can be drawn that DARPin-based CAR-T therapy does not concede to T lymphocytes that carry artificial receptors containing single-chain antibodies, and the comparative simplicity of obtaining DARPins and their monomeric form facilitates the creation of chimeric receptors for different targets.

Natural killer (NK) cells can also be utilized as agents for tumor recognition using chimeric antigen receptors. Their cytotoxicity is based on the same mechanisms as the activity of CD8^+^ T cells; the natural activation pathways provide some advantages to CAR-NK over CAR-T. NK cells do not recognize a peptide in complex with MHC I [101], which reduces the risk of graft-versus-host disease (GVHD). This feature has already been used in cancer therapy by transfusion of donor NK cells [102, 103, 104] or even cells of the stable NK-92 line [105, 106], which increases therapy effectiveness even without using a chimeric antigen receptor. This makes it possible to design therapy based on stable NK cell lines that does not require cells from the patient [107]. Additional benefits of NK cells include their natural mechanisms of damaged cell recognition, which allows them to remain efficient antitumor agents even if the chimeric antigen receptor gene is lost or mutant. To date, no antitumor CAR-NK therapy based on DARPins has been developed, but it probably will soon be elaborated.

## CONCLUSIONS

DARPins were designed as scaffold proteins alternative to antibodies. They are used in most technologies that originally utilize antibodies, except for those technologies where the properties of the constant part of immunoglobulin molecules are needed. The advantages of DARPins, including their small size, independence of animal immunization, and simplicity of production of fusion proteins, make them promising tools for research and efficient components of therapeutic and diagnostic agents. One should refrain from a conclusion that alternative scaffolds can completely replace antibodies; however, they surely have made a substantial contribution to the targeting proteins being utilized and expanded the range of possible targets due to the different paratope structure. Furthermore, they have provided exceptional opportunities for creating bispecific and multivalent constructs.

## Abbreviations

DARPin: designed ankyrin repeat protein
scFv: single-chain variable fragment of an antibody
HER2: human epidermal growth factor receptor 2
EGFR: epidermal growth factor receptor;
EpCAM: epidermal growth factor receptor
IgE: immunoglobulin E
